# EZH2-Associated Hypermethylated Gene Signature Predicts Immunotherapy Response and Implicates DUSP5 in Tumor-Immune Regulation in Triple-Negative Breast Cancer

**DOI:** 10.3390/cancers18101606

**Published:** 2026-05-15

**Authors:** Mingzhan Xue, Sujitha Jeya, Reem Elasad, Sarra Mestiri, Fares Al Ejeh, Mariam Al-Muftah

**Affiliations:** 1 Translational Oncology Research Centre, Qatar Biomedical Research Institute, Hamad Bin Khalifa University, Doha P.O. Box 34110, Qatar; mxue@hbku.edu.qa (M.X.); sjeya@hbku.edu.qa (S.J.); relasad01@qub.ac.uk (R.E.); smestiri@hbku.edu.qa (S.M.); falejeh@hbku.edu.qa (F.A.E.); 2 College of Health and Life Sciences, Hamad Bin Khalifa University, Doha P.O. Box 34110, Qatar

**Keywords:** Triple-Negative Breast Cancer, EZH2, DNA methylation, immunotherapy, DUSP5, MAPK signaling, biomarker, immune regulation

## Abstract

**Background/Objectives:** Triple-negative breast cancer (TNBC) is a candidate for immune checkpoint blockade; however, current biomarkers remain insufficient to predict therapeutic response or capture tumor-intrinsic mechanisms. Enhancer of Zeste Homolog 2 (EZH2)-mediated epigenetic repression has been implicated in immune evasion, yet the contribution of EZH2-repressed genes to anti-tumor immunity and clinical outcomes in TNBC remains unclear. We aimed to identify EZH2-associated epigenetically repressed genes in TNBC and evaluate their relevance as tumor-intrinsic regulators and potential predictors of immunotherapy outcome. **Methods:** We performed integrative in silico analyses of The Cancer Genome Atlas (TCGA) breast cancer cohorts to identify EZH2-associated hypermethylated genes in TNBC. A composite 30-gene signature (30GS) was defined based on transcriptional repression and promoter hypermethylation. Associations with clinical outcomes, tumor- and immune-related programs, and therapeutic response were evaluated, with validation in the I-SPY2 cohort and an independent TNBC patient cohort. **Results:** The 30GS was significantly reduced in TNBC and basal-like tumors and associated with improved clinical outcomes and enrichment of tumor- and immune-related signatures. In the I-SPY2 cohort, the 30GS predicted pathological complete response in patients receiving chemo-immunotherapy (AUC = 0.7377, *p* = 0.0007). Gene-level analysis identified Dual Specificity Phosphatase 5 (DUSP5) as the gene most consistently associated with immune-related parameters. In an independent TNBC cohort, DUSP5-high tumors demonstrated transcriptional programs enriched for inflammatory, immune-related, and signaling pathways within the NanoString Breast Cancer 360 panel. **Conclusions:** This study defines an EZH2-associated epigenetic program linked to tumor-intrinsic immune programs in TNBC and identifies DUSP5 as a candidate gene associated with immune-related transcriptional states.

## Simple Summary

Triple-negative breast cancer (TNBC) is an aggressive form of breast cancer that lacks reliable tools to predict which patients will benefit from immunotherapy. In this study, we investigated how chemical modifications that control gene activity may influence the immune response in TNBC. We identified a group of 30 genes that are switched off in TNBC and found that their combined activity is linked to better patient outcomes and better response to immunotherapy. Among these genes, DUSP5 stood out as being strongly associated with an ‘immune-active’ environment within the tumor. These findings suggest that measuring changes in these genes, particularly DUSP5, could help identify patients more likely to benefit from immunotherapy and guide future treatment strategies.

## 1. Introduction

Triple-negative breast cancer (TNBC), defined by the absence of estrogen receptor (ER), progesterone receptor (PR), and human epidermal growth factor receptor 2 (HER2) amplification/overexpression, accounts for approximately 15–20% of invasive breast cancers [1]. TNBC remains a clinically challenging subtype due to its aggressive behavior characterized by early recurrence, a high risk of distant metastasis, resistance to therapy, and poor clinical outcomes [2,3]. Biologically, TNBC is considered an immunologically active subtype, exhibiting increased immune cell infiltration within the tumor microenvironment, higher tumor mutational burden (TMB), and elevated expression of immune checkpoint molecules such as programmed death ligand-1 (PD-L1) [4,5,6]. In recent years, immune checkpoint inhibitors targeting programmed cell death protein 1 (PD-1) and PD-L1, particularly when combined with chemotherapy, have demonstrated improved clinical outcomes in subsets of early-stage and metastatic TNBC patients compared with conventional chemotherapy alone [7,8]. Nevertheless, therapeutic efficacy remains suboptimal, with a substantial proportion of patients failing to respond and many exhibiting primary or acquired resistance, reflecting significant heterogeneity in treatment outcomes [8].

Despite the clinical integration of immune checkpoint inhibitors in TNBC, reliable biomarkers for patient stratification and prediction of therapeutic response remain limited. Currently, PD-L1 expression is used to guide patient selection in certain clinical settings; however, it is dynamic, context-dependent, and influenced by treatment, with reduced predictive value, particularly in the metastatic setting. Responses have been observed in both PD-L1-positive and PD-L1-negative patients, underscoring its inconsistency as a standalone biomarker [9]. Similarly, tumor-infiltrating lymphocytes and immune-related gene expression signatures have been associated with improved response to immunotherapy and favorable prognosis; however, these biomarkers lack standardization, specificity, and reproducibility for reliable clinical stratification [10,11]. Genomic features such as TMB, while associated with immunogenicity, have shown limited predictive accuracy in TNBC [5]. Importantly, these biomarkers primarily reflect the immune landscape of the tumor microenvironment and do not fully capture tumor-intrinsic programs that regulate immune activation and immune evasion. This highlights a critical gap in understanding how cancer cell-intrinsic pathways shape the immunogenicity of TNBC and may influence response to immune checkpoint blockade.

Epigenetic regulation, particularly DNA methylation, has emerged as a critical determinant of tumor-intrinsic mechanisms influencing tumor progression, anti-tumor immune responses, and therapeutic outcomes in TNBC, with increasing evidence demonstrating that epigenetic remodeling contributes to immune evasion and therapeutic response [12,13,14]. Among epigenetic modifiers, Enhancer of Zeste Homolog 2 (EZH2), the catalytic subunit of the Polycomb Repressive Complex 2 (PRC2), is frequently upregulated in TNBC and mediates transcriptional repression through trimethylation of histone H3 lysine 27 [15,16]. Beyond its established role in tumor proliferation and metastasis, accumulating evidence indicates that EZH2-driven transcriptional silencing affects genes involved in antigen presentation, interferon signaling, and chemokine production [12,17]. Consistent with this, epigenetic repression of immune-related genes has been implicated in tumor immune evasion and resistance to immune checkpoint blockade [18,19]. Given the role of EZH2 in shaping anti-tumor immunity in TNBC, EZH2-regulated genes may represent a class of tumor-intrinsic regulators with the potential to influence and predict responses to immune checkpoint blockade.

Among potential EZH2-regulated genes, Dual Specificity Phosphatase 5 (DUSP5) is a nuclear phosphatase that selectively dephosphorylates extracellular signal-regulated kinases 1 and 2 (ERK1/2), thereby negatively regulating the mitogen-activated protein kinase (MAPK) pathway [20]. The MAPK/ERK pathway plays a central role in tumor cell proliferation, survival, and invasion, and has also been implicated in regulating immune-related processes, including interferon signaling, cytokine production, and expression of immune checkpoint molecules [21,22]. While the role of DUSP5 in tumor immune regulation remains largely unexplored, recent evidence suggests that loss of DUSP5 promotes MAPK activation and TNBC tumor progression, consistent with its function as a negative regulator of ERK signaling [23]. Given this, epigenetic repression of EZH2 target genes, including DUSP5, may contribute to aberrant signaling associated with immune evasion and altered response to immune checkpoint blockade. In this study, we systematically investigated EZH2-associated hypermethylated genes in TNBC to identify tumor-intrinsic immune-related programs and potential biomarkers associated with immunotherapy response. Based on integrative analyses across independent cohorts, DUSP5 was subsequently prioritized for further investigation as a candidate gene associated with tumor-related transcriptional programs in TNBC.

## 2. Materials and Methods

### 2.1. TCGA Breast Cancer Dataset and Subtype Classification

Gene expression and DNA methylation data for breast cancer were obtained from The Cancer Genome Atlas (TCGA) breast cancer (BRCA) cohort. Cases were stratified into breast cancer subtypes based on immunohistochemistry (IHC) classification, including triple-negative breast cancer (TNBC), hormone receptor-positive/HER2-negative (HR+/HER2-), and hormone receptor-negative/HER2-positive (HR-/HER2+). For analyses involving matched adjacent normal tissue, a subset of cases with available paired tumor and adjacent normal samples was used (*n* = 103 tumor-normal pairs; 13 TNBC, 25 HR-/HER2+, and 65 HR+/HER2-). For subtype comparison analysis, the full TCGA BRCA cohort was used (TNBC, *N* = 165; HR-/HER2+, *n* = 176; HR+/HER2-, *n* = 622).

#### 2.1.1. Unsupervised Clustering and Principal Component Analysis

Unsupervised hierarchical clustering was performed using normalized gene expression data from tumor and matched adjacent normal tissues to assess global transcriptomic differences. Clustering was conducted in R using the gplots package (version 3.1.1). Principal component analysis (PCA) was performed using the prcomp function in the stats R package (version 4.1.1) to visualize the separation between tumor and adjacent normal samples, as well as among breast cancer cell lines. Volcano plots were generated using iDEP.92 (version 0.92) installed in RStudio (version 1.2.5).

#### 2.1.2. Differential Gene Expression Analysis

Differential gene expression analysis was performed using the limma package (version 3.28.14) to compare tumor samples and their matched adjacent normal tissues within each breast cancer subtype. Expression of lysine-specific histone methyltransferases (KMTs) across breast cancer subtypes was compared using two-way ANOVA with Dunnett’s multiple comparisons. Expression ratios (tumor versus normal) were calculated for each gene. To identify EZH2-associated transcriptional changes, differentially expressed genes (DEGs) were intersected with a curated list of 1940 EZH2 target genes obtained from the ChIP Enrichment Analysis (ChEA) dataset [24]. DEGs were classified as upregulated or downregulated relative to adjacent normal tissue. For subtype-specific analyses, expression of EZH2-target genes was compared between TNBC and other subtypes (HR+/HER2- and HR-/HER2+). Genes significantly downregulated in TNBC relative to both subtypes were identified by intersecting the respective DEG lists.

#### 2.1.3. DNA Methylation Analysis and Correlation with EZH2 Expression

DNA methylation levels were analyzed using β-values for each gene. Average methylation levels were calculated for each breast cancer subtype. Genes were classified as hypermethylated or hypomethylated in TNBC relative to other subtypes. To assess the relationship between EZH2 expression and DNA methylation, a Pearson correlation coefficient was calculated between EZH2 mRNA expression levels and gene-specific methylation across the TCGA cohort. Statistical significance of the correlation was determined using two-tailed *p*-values.

#### 2.1.4. Calculation of the 30-Gene Signature Score (30GS)

The 30-gene signature (30GS) score was calculated as the unweighted average normalized expression of the following genes: DUSP5, SLC7A8, PGR, SCN7A, APOF, MSX2, CPEB2, NCAM2, DUSP4, KCNC2, PTGER3, TFAP2B, CAMK2N1, ST8SIA6, GATA3, LRIG1, SCUBE2, SIDT1, RND1, BCL2, TM7SF2, CCDC30, LFNG, AR, ADAMTS15, SOWAHA, MAPT, NTN4, PTPRT, and EFCC1.

#### 2.1.5. Survival Analysis

Patients were stratified into tertiles (low, intermediate, and high) based on the distribution of the 30GS score within the TGCA BRCA cohort. The lowest tertile represented low 30GS expression, while the middle and high tertiles were grouped as high 30GS expression. Survival analyses were performed in GraphPad Prism (Version 11) to evaluate associations between 30GS and clinical outcomes, including breast cancer disease-specific survival (DSS), progression-free intervals (PFI), disease-free intervals (DFI), and distant metastasis-free survival (DMFS). Kaplan–Meier survival curves were generated, and statistical significance was assessed using the log-rank test. Hazard ratios (HRs) and corresponding 95% confidence intervals were calculated. Multivariable Cox proportional hazards analyses were performed to evaluate the association between the 30GS and different survival endpoints, adjusting for age and pathological stage. HRs and 95% confidence intervals were visualized using forest plots generated in R.

#### 2.1.6. 30GS Association Analysis with Tumor- and Immune-Related Transcriptional Programs

The 30GS score was correlated with previously published tumor- and immune-related transcriptional signatures derived from breast cancer datasets and applied within the TCGA cohort to characterize the tumor immune landscape. Spearman correlation coefficients (r) were calculated in GraphPad Prism between the 30GS and each transcriptional signature. Gene-level analysis was performed by calculating r between individual genes within the 30GS and the same transcriptional signatures.

### 2.2. I-SPY2 Patient Cohort and Data Analysis

Clinical data and gene expression data from pre-treatment tumor biopsies were obtained from the I-SPY2 clinical trials cohort (GEO accession: GSE194040). Gene expression profiling was performed using Agilent 44K whole-transcriptome microarrays [25]. In our study, gene expression data were obtained as log2-transformed and normalized expression values, which were used directly for downstream analyses. To calculate the 30GS score, expression of *ST8SIA6* was not available in the I-SPY2 dataset; therefore, the score was calculated using the same unweighted average approach with the remaining available 29 genes, while maintaining the original signature definition. Patients were stratified according to breast cancer subtypes, including HR+/HER2- (*n* = 379), HR+/HER2+ (*n* = 156), HR-/HER2+ (*n* = 89), and TNBC (*n* = 364), irrespective of treatment arm. Differences across subtypes were assessed using one-way ANOVA.

For TNBC-specific analyses, patients were further classified using Lehmann classifications [26] (basal-like 1 [BL1, *n* = 60], basal-like 2 [BL2, *n* = 28], immunomodulatory [IM, *n* = 96], luminal androgen receptor [LAR, *n* = 26], mesenchymal [M, *n* = 67]) and mesenchymal stem-like [MSL, *n* = 33] and Burstein classification [27] (basal-like immune activated [BLIA, *n* = 92], basal-like immune suppressed [BLIS, *n* = 108], luminal androgen receptor [LAR, *n* = 80], and mesenchymal [MES, *n* = 72]).

Pathological complete response (pCR) was used as the primary clinical endpoint. TNBC patients were stratified based on treatment group, paclitaxel (*n* = 85) and paclitaxel plus pembrolizumab (*n* = 29). Receiver operating characteristic (ROC) analysis was performed to evaluate predictive performance. The area under the curve (AUC) and corresponding *p*-value were calculated. To benchmark predictive performance, ROC analysis was also performed using previously established predictors of response to immunotherapy [25], including dendritic_cells, ICS5_score, Module Tcell/Bcell score, chemokine 12_score, and STAT_sig. Gene-level correlation analysis was performed using Pearson correlation between expression of individual genes within the 30GS and predictors of response to immunotherapy.

### 2.3. TNBC Patient Cohort and NanoString Gene Expression Analysis

Formalin-fixed paraffin-embedded (FFPE) tumor tissues from 34 patients diagnosed with TNBC were purchased from TriStar Technology Group (https://tristargroup.us). Inclusion criteria included estrogen receptor-negative, progesterone receptor-negative, and HER2-negative status as determined by IHC and histologically confirmed invasive ductal carcinoma. Clinicopathological characteristics of the cohort are summarized in Supplementary Table S1. Among the cohort, 14 samples were treatment-naïve (pre-treatment), and 20 samples were obtained from patients treated with neoadjuvant epirubicin and cyclophosphamide (post-treatment). Total RNA was extracted using the All Prep FFPE Kit (QIAGEN, Hilden, Germany; Cat no. 80234) following the manufacturer’s protocols. RNA concentration was assessed using Qubit fluorometry, and RNA integrity and fragment distribution were evaluated using the Agilent Bioanalyzer to ensure suitability for downstream applications. DUSP5 expression was quantified by quantitative PCR (qPCR), and ∆CT values were calculated relative to the average CT of two housekeeping genes (*PRL19* and *ACTB*). Relative expression was computed using the 2^−∆CT^ method and was log2-transformed. Tumors were stratified into DUSP5-high (DUSP5 H) and DUSP5-low (DUSP5 L) groups based on the upper and lower 50% of DUSP5 expression.

Gene expression profiling was performed using the NanoString nCounter Breast Cancer 360 (BC360) panel (NanoString Technologies, Seattle, WA, USA). Raw counts were normalized using the nSolve software (Version 4.0) in accordance with the manufacturer’s guidelines.

Differential gene expression analysis comparing DUSP5 H and DUSP5 L tumors within the pre-treatment and post-treatment cohorts, as well as pre-treatment versus post-treatment tumors, was performed using the limma pipeline in R as described above. To evaluate the association between DUSP5-associated transcriptional programs and clinical outcomes, survival analyses were performed independently within the pre-treatment and post-treatment cohorts. Differential expression analyses comparing tumors from patients who were alive versus those who were dead, and Cox proportional hazards regression analyses, were performed as described above. Heatmaps were generated in R using log2 fold-change values and significance from differential expression analyses. Pathway analysis of DUSP5-associated genes and survival-associated subsets was performed using STRING-DB (https://string-db.org/) with KEGG pathway annotation.

## 3. Results

### 3.1. EZH2 Is Preferentially Upregulated in TNBC and Associated with Enrichment of EZH2 Transcriptional Targets

**Figure 1:**
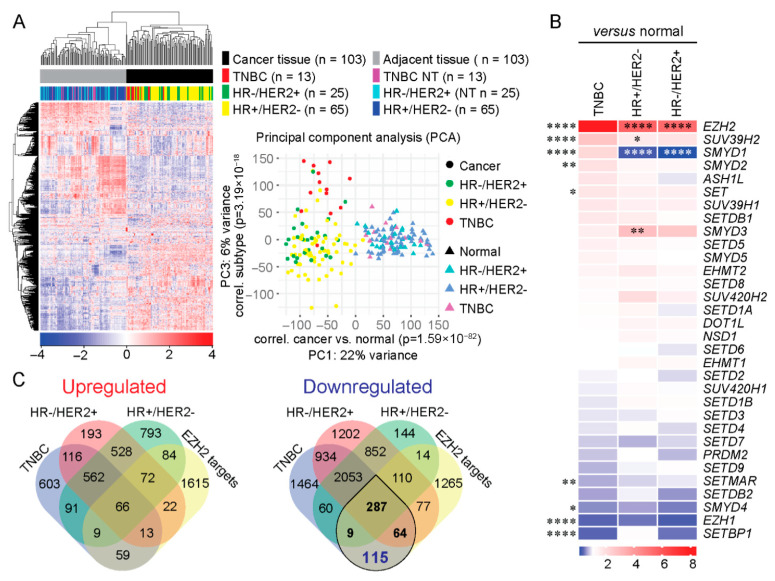
EZH2 is preferentially upregulated in TNBC and associated with enrichment of its transcriptional targets. Breast cancer cases with matched adjacent normal tissue from The Cancer Genome Atlas (TCGA) breast cancer dataset were used for the analysis. The cohort comprised 13 TNBC, 25 HR-/HER2+, and 65 HR+/HER2- cases. (**A**) Unsupervised hierarchical clustering and principal component analysis (PCA) of 103 breast cancer tumors and their matched adjacent normal breast tissues based on global transcriptomic profiles. (**B**) Differential mRNA expression of lysine-specific histone methyltransferases (KMTs) across breast cancer subtypes relative to matched adjacent normal tissue. Data are presented as tumor-to-normal expression ratios. Asterisks on the left indicate statistical significance of expression differences between TNBC and adjacent normal tissue. Asterisks within the heatmap indicate significant differences in expression ratios between TNBC and other subtypes. Statistical analysis was performed using two-way ANOVA with Dunnett’s multiple comparison tests (* *p* < 0.05, ** *p* < 0.01, **** *p* < 0.0001). (**C**) Venn diagrams showing the overlap between differentially expressed genes (DEGs) in each breast cancer subtype relative to adjacent normal tissue and a curated list of 1940 EZH2 target genes from the ChEA dataset. Upregulated and downregulated DEGs are shown in the left and right panels, respectively. Downregulated genes in TNBC that overlap with EZH2 targets are marked.

To investigate subtype-specific epigenetic alterations within breast cancer, we performed unsupervised hierarchical clustering of tumor samples and matched adjacent normal breast tissues from The Cancer Genome Atlas (TCGA) breast cancer cohort. Unsupervised analysis demonstrated clear segregation of tumor tissue from adjacent normal breast tissues (Figure 1A), confirming distinct global transcriptional profiles. Within tumor tissues, breast cancer subtypes exhibited partial segregation. TNBC cases clustered more distinctly from other breast cancer subtypes, particularly ER+/HER2- tumors, consistent with a subtype-specific transcriptional program.

Next, we examined the expression of lysine-specific histone methyltransferases (KMTs) across breast cancer subtypes relative to matched normal tissue. Differential expression analysis revealed several subtype-specific dysregulated KMT genes, with EZH2 demonstrating the greatest degree of upregulation across subtypes (Figure 1B). EZH2 expression was markedly upregulated in TNBC (ratio 8.5 ± 1.7, *p* < 0.0001), ER+/HER2- tumors (ratio 5.6 ± 0.9, *p* < 0.0001), and HR-/HER2+ tumors (ratio 5.3 ± 1.0, *p* < 0.0001) compared to adjacent normal breast tissues. Importantly, the magnitude of EZH2 upregulation in TNBC was significantly higher than in HR+/HER2- and HR-/HER2+ subtypes (*p* < 0.0001), highlighting a preferential upregulation of EZH2 in TNBC.

To determine whether EZH2 overexpression in TNBC is associated with enrichment of its downstream transcriptional program, we intersected subtype-specific differentially expressed genes (DEGs) with a curated list of 1940 EZH2 target genes derived from the ChEA dataset (ChIP Enrichment Analysis) [24]. Overlap analysis revealed greater overlap between TNBC-associated DEGs and EZH2 target genes compared with other breast cancer subtypes (Figure 1C). Out of the 1940 EZH2-target genes, 325 (16.7%) were upregulated, and 676 (34.8%) were downregulated across the three breast cancer subtypes. Among the 475 downregulated TNBC-associated EZH2 target genes, 287 were shared across breast cancer subtypes, whereas 9 and 64 genes were shared exclusively with HR+/HER2- and HR-/HER2+ tumors, respectively. A total of 115 downregulated EZH2 target genes were uniquely dysregulated in TNBC, indicating a TNBC-associated EZH2-linked transcriptional signature.

### 3.2. Identification of a TNBC-Associated EZH2-Linked Hypermethylated Gene Signature

**Figure 2:**
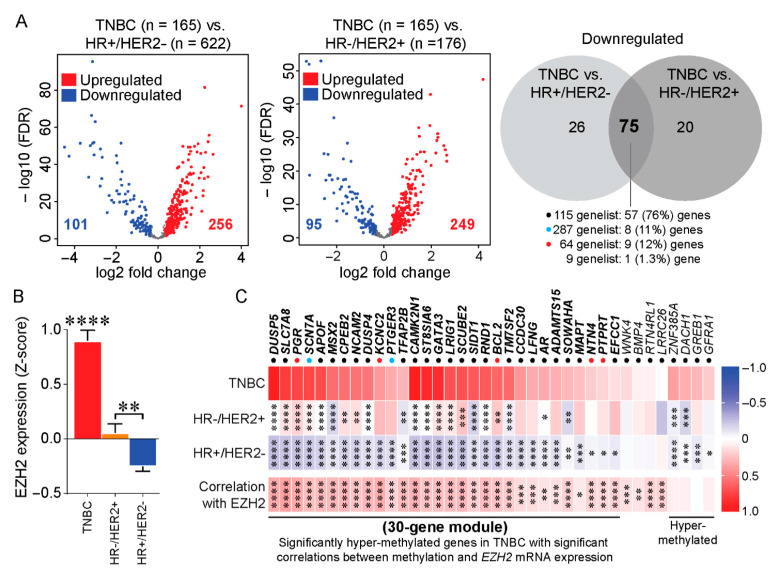
Identification of a TNBC-associated EZH2-linked hypermethylated gene signature. A subset of 475 EZH2 target genes that were downregulated in TNBC relative to adjacent normal tissue were further analyzed across the TCGA breast cancer cohort. (**A**) Differential expression analysis of the 475 EZH2 target genes comparing TNBC (*n* = 165) to HR+/HER2- (*n* = 622) and HR-/HER2+ (*n* = 176) breast cancer subtypes. A total of 101 and 95 genes were identified as significantly downregulated in TNBC relative to HR+/HER2- and HR-/HER2+ tumors, respectively. Intersections of these gene sets identified 75 genes commonly downregulated in TNBC compared to both subtypes. (**B**) EZH2 mRNA expression was determined as a z-score across breast cancer subtypes. EZH2 expression levels are shown for TNBC, HR+/HER2-, and HR-/HER2+ tumors. One-way ANOVA was used for statistical comparison. (**C**) DNA methylation analysis of the 75 commonly downregulated EZH2 target genes across breast cancer subtypes and correlation with EZH2 mRNA expression. Methylation levels were presented as average beta values for each subtype (red: hypermethylated; blue: hypomethylated). Statistical comparisons between subtypes were performed using two-way ANOVA and Dunnett’s multiple comparisons tests (* *p* < 0.05, ** *p* < 0.01, *** *p* < 0.001, **** *p* < 0.0001). Correlation between gene-specific methylation and EZH2 mRNA expression across the TCGA cohort was assessed using a two-tailed Pearson correlation, with the correlation coefficient (r) and corresponding *p*-values indicated (lower panel). Colored dots beneath each gene indicate their origin from Figure 1C: black dots represent genes uniquely downregulated in TNBC, blue dots represent genes commonly downregulated across all breast cancer subtypes, and red dots represent genes shared between TNBC and HR-/HER2+ subtypes. Remaining genes from the 75-gene set are shown in Supplementary Figure S1.

To define EZH2-specific transcriptional programs that distinguish TNBC from other breast cancer subtypes, we performed differential expression analysis of 475 EZH2 target genes dysregulated in TNBC (*n* = 165) compared to HR+/HER2- (*n* = 622) and HR-/HER2+ (*n* = 176) tumors in the TCGA breast cancer cohort. Among these genes, 101 genes were downregulated in TNBC relative to HR+/HER2- tumors, and 95 genes were downregulated in TNBC relative to HR-/HER2+ tumors (Figure 2A). The intersection of these datasets identified 75 genes that were commonly downregulated in TNBC relative to both subtypes. Notably, 57 of these 75 genes (76%) originated from the subset of 115 genes uniquely downregulated in TNBC relative to adjacent normal breast tissue (Figure 1C), supporting the presence of a transcriptionally repressed TNBC-associated program.

To further refine this gene set, we examined whether repression of these 75 EZH2 target genes was associated with elevated EZH2 expression and increased promoter hypermethylation. Consistent with our earlier findings, EZH2 mRNA levels were significantly higher in TNBC tumors compared to HR+/HER2- and HR-/HER2+ tumors (Figure 2B), further supporting preferential EZH2 upregulation in TNBC. Assessment of DNA methylation revealed that 30 of the 75 genes (40%) were significantly hypermethylated in TNBC compared with other breast cancer subtypes (Figure 2C). Importantly, methylation levels of these genes positively correlated with EZH2 mRNA expression across the TCGA cohort, indicating an association between elevated EZH2 expression, increased DNA methylation, and transcriptional repression of these genes. In contrast, 25 of the 75 genes (33.3%) were significantly hypomethylated in TNBC compared to other breast cancer subtypes, despite being transcriptionally downregulated (Supplementary Figure S1). This observation indicates that repression of this subset of genes may involve mechanisms beyond EZH2-associated methylation. Collectively, these analyses identified a TNBC-enriched 30-gene signature characterized by transcriptional repression and promoter hypermethylation in association with elevated EZH2 expression, consistent with an epigenetically associated silencing program in TNBC.

### 3.3. Clinical Relevance of the TNBC-Associated 30-Gene EZH2-Linked Hypermethylated Signature

**Figure 3:**
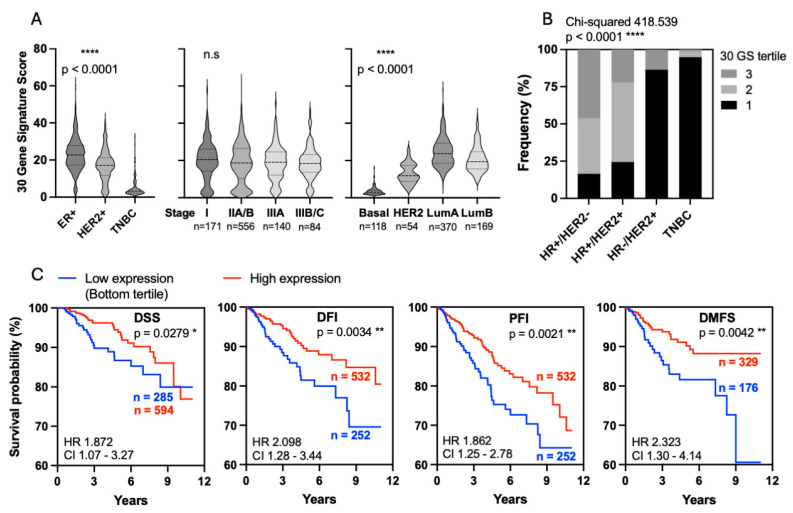
Clinical relevance of the TNBC-associated 30-gene signature. (**A**) A set of 30 EZH2 target genes that were hypermethylated and downregulated in TNBC relative to adjacent normal tissue and other breast cancer subtypes was used to calculate a composite gene expression score (30GS) for each case in the TCGA breast cancer dataset. Shown is the distribution of the 30GS across breast cancer subtypes and clinical stage. The 30GS is lower in TNBC tumors compared to HR+/HER2- and HR-/HER2+ subtypes, PAM50 molecular subtypes, and pathological stages. Statistical analysis was performed using one-way ANOVA with Tukey’s multiple comparison test (*p* < 0.0001 for all comparisons except disease stage). (**B**) Distribution of patients across 30GS tertiles within each breast cancer subtype. Frequencies of cases within each tertile (low, intermediate, and high) are shown for TNBC, HR+/HER2-, HR+/HER2+, and HR-/HER2+. Statistical analysis was performed using the chi-squared test, and corresponding *p*-values are indicated. (**C**) Association of the 30GS with clinical outcome. Patients were stratified based on the 30GS tertiles, and Kaplan–Meier analyses were performed for breast cancer-specific survival (BCSS), disease-free interval (DFI), progression-free interval (PFI), and distant metastasis-free survival (DMFS). Hazard ratios (HR), 95% confidence intervals, and *p*-value were calculated using the log-rank test. n.s = not significant, * *p* < 0.05, ** *p* < 0.01 and **** *p* < 0.0001.

To evaluate the clinical and biological significance of the TNBC-enriched 30-gene EZH2-associated hypermethylated signature, we calculated a composite expression score (metagene score) defined as the average normalized expression of the 30 genes. The 30-gene signature (30GS) score was significantly downregulated in TNBC tumors compared to HR+/HER2- and HR-/HER2+ subtypes (Figure 3A). Consistent with this finding, stratification by intrinsic molecular subtype demonstrated significantly lower 30GS scores in basal-like tumors compared to Luminal A, Luminal B, and HER2-enriched tumors. No significant differences in the 30GS were observed across pathological stages (I, IIA/B, IIIA, and IIIB/C), consistent with subtype-associated rather than stage-associated repression of this gene set.

To determine the prognostic value of the 30GS, tumors were stratified into tertiles based on 30GS expression levels. A substantial proportion of TNBC (94.9%) and HR-/HER2+ (86.5%) patients were classified in the lowest tertile of 30GS expression, whereas 16.6% of HR+/HER2- tumors fell within this tertile (Figure 3B). Patients with 30GS-high tumors in the TCGA breast cancer dataset had significantly improved clinical outcomes across multiple survival endpoints, including disease-specific survival (DSS), disease-free interval (DFI), progression-free interval (PFI), and distant metastasis-free survival (DMFS) (Figure 3C). The strongest survival separation was observed in DMFS, indicating a potential association between reduced 30GS and increased metastatic risk. Multivariable survival analysis demonstrated that intermediate- and higher- 30GS groups were consistently associated with reduced risk of clinical events across different endpoints (DMFS, DSS, and DFI) compared to the lowest 30GS group, while higher pathological stage was associated with increased risk, consistent with independent associations of 30GS expression and pathological stage with clinical outcome (Supplementary Figure S2).

### 3.4. The TNBC-Associated 30-Gene Signature Is Associated with Immune-Related Transcriptional Programs in TNBC

**Figure 4:**
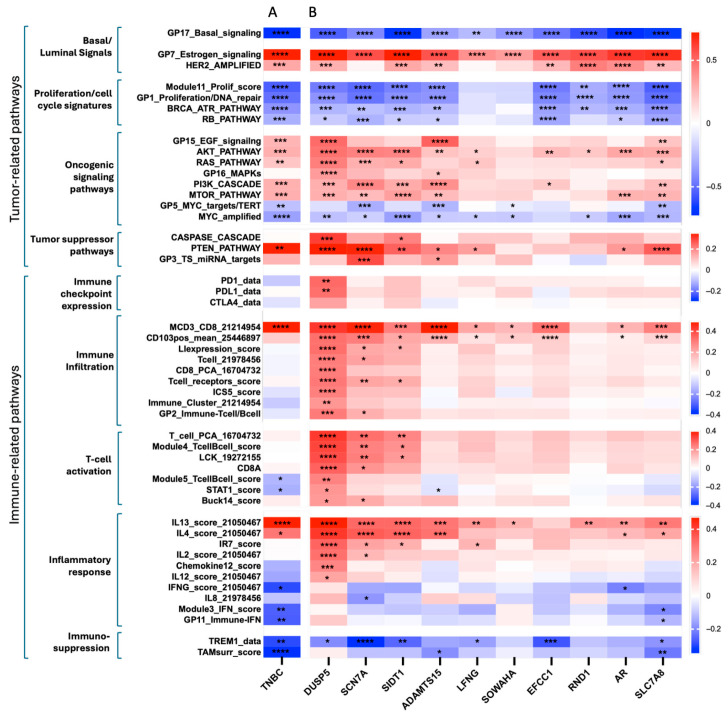
Association of the 30-gene signature with tumor- and immune- related transcriptional programs in TNBC. (**A**) Correlation of the 30-gene score (30GS) with tumor- and immune-related transcriptional signatures in TNBC cases from the TCGA breast cancer dataset. The heatmap values represent Pearson correlation coefficients (r), with corresponding two-tailed *p*-values indicated (* *p* < 0.05, ** *p* < 0.01, *** *p* < 0.001 **** *p* < 0.0001). (**B**) Gene-level correlation analysis between individual genes within the 30GS and tumor-and immune- related transcriptional signatures in TNBC cases from the TCGA breast cancer dataset. Correlation coefficients (r) were calculated using Pearson correlation with corresponding two-tailed *p*-values indicated. Data are shown for the top 10 significantly associated genes.

Given the role of epigenetic silencing in tumor immune evasion, we next investigated the association between the 30GS score and tumor- and immune-related transcriptional programs in TNBC. Correlation analysis revealed that the 30GS is strongly associated with tumor-intrinsic biological pathways (Figure 4A). Specifically, the 30GS demonstrated strong inverse correlations with basal-like and proliferative programs, including GP17_Basal signaling (r = −0.77, *p* < 0.0001), Module11_proliferation score (r = −0.57, *p* < 0.0001), and GP1_Proliferation/DNA repair (r = −0.51, *p* < 0.0001), consistent with an association between lower 30GS expression and aggressive tumor phenotypes. In contrast, the 30GS was positively correlated with luminal-associated pathways, including estrogen signaling (r = 0.78, *p* < 0.0001), as well as HER2-related and PI3K/AKT/mTOR signaling pathways, supporting its association with less aggressive tumor states. With respect to immune-related pathways, the 30GS demonstrated selective rather than global associations with the tumor immune landscape. A positive correlation was observed with the CD8-related signature (r = 0.33, MCD3_CD8, *p* < 0.0001), suggesting a link with T-cell infiltration. In contrast, interferon-related pathways (Module3_IFN, GP11_Immune_IFN) and myeloid-association signatures (TAMsurr_score) exhibited significant inverse correlations with the 30GS. These findings suggest that the 30GS primarily reflects tumor-intrinsic biological states, with context-dependent associations with specific immune pathways rather than a uniform immune activation profile.

To further dissect the biological relevance of individual genes within the 30GS, specifically in TNBC, we performed gene-level correlation analysis exclusively in TNBC tumors against established tumor and immune-related pathway signatures (Figure 4B). Collectively, the genes inversely correlated with basal-like signaling programs and positively correlated with estrogen- and HER2-associated signatures, consistent with a luminal-associated transcriptional program selectively repressed in TNBC. Many genes demonstrated significant inverse correlations with proliferation- and cell-cycle-related pathways, including Module11_Proliferation score, GP1_Proliferation/DNA_repair, BRCA_ATR_Pathway, and PB_Pathway. These associations suggest that repression of these genes may contribute to enhanced proliferation capacity and tumor progression in TNBC.

With respect to immune-related signatures, the genes showed broad and significant associations with multiple immune parameters. Specifically, they positively correlated with immune checkpoint-related signatures (PD-1, PD-L1, and CTLA-4), immune infiltration signatures (MCD3_CD8, CD103pos_mean, LIexpression_score, Tcell, CD8_PCA, Tcell_receptor_score, ICS5_score, Immune_clusters, and GP2_immune_Tcell/Bcell), and T-cell activation signatures (T_cell_PCA, Module4_Tcell/Bcell_score, LCK, CD8A, Module5_Tcell/Bcell, STAT1 and Buck14_score). In addition, the genes were positively correlated with inflammatory response signatures (IL13, IL14, IR7, IL2, Chemokine12, IL12, IFNG, IL8, Module3_IFN, and GP11_immuneIFN scores), and negatively correlated with immunosuppressive signatures (TERM1 and TAMsurr_score). Among the genes, DUSP5 emerged as the most significantly associated gene across multiple tumor- and immune-related parameters, demonstrating particularly strong correlations with immune infiltration and T-cell activation signatures. These findings identify DUSP5 as a candidate gene linking EZH2-associated transcriptional repression with immune-related signatures in TNBC. While these correlation analyses cannot distinguish whether these immune-associated patterns reflect an enhanced anti-tumor immune response or adaptive immune evasion, several associations with checkpoint and immune modules suggest that repression of genes within the 30GS, including DUSP5, is associated with immune-related transcriptional states in TNBC.

### 3.5. Independent Validation of 30-Gene Signature in the I-SPY2 Neoadjuvant Cohort

**Figure 5:**
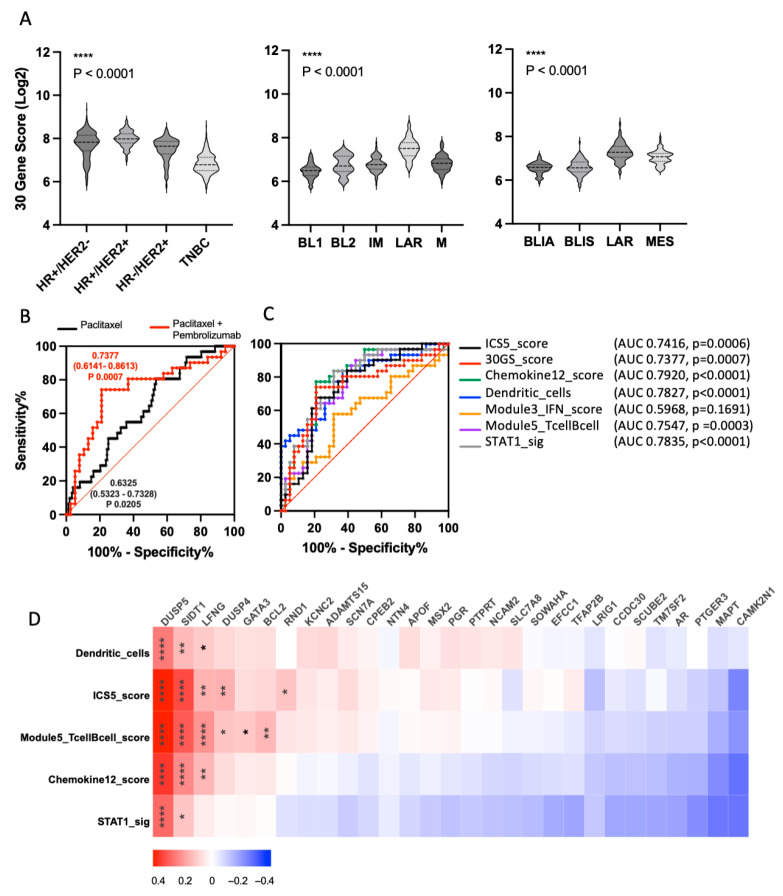
Independent validation of the 30-gene signature in the I-SPY2 cohort and association with treatment response. (**A**) Distribution of the 30-gene signature (30GS) across breast cancer subtypes and TNBC molecular classifications in the I-SPY2 cohort. The 30GS is shown across breast cancer subtypes (HR+/HER2-, HR+/HER2+, HR-/HER2+, and TNBC), as well as TNBC subtypes defined by the Lehmann classification (BL1, BL2, IM, LAR, M) and Burstein classification (BLIA, BLIS, LAR, MES). Statistical analysis was performed using ordinary one-way ANOVA, with all subgroup comparisons showing *p* < 0.0001. (**B**) Receiver operating characteristic (ROC) analysis evaluating the association of the 30GS with pathological complete response (pCR) in TNBC patients treated with paclitaxel alone or in combination with pembrolizumab. Area under the curve (AUC) and corresponding *p*-value are indicated. (**C**) ROC analysis comparing the performance of the 30GS with previously reported immune-related predictors from the I-SPY2 clinical trial in TNBC patients treated with paclitaxel and pembrolizumab. AUC values, standard error, 95% confidence intervals (CI), and *p*-values are shown for each predictor. (**D**) Gene-level correlation analysis between individual genes within the 30GS and previously reported predictors of immunotherapy response in TNBC patients across all treatment arms (*n* = 363). Pearson correlation coefficient (r) and corresponding *p*-values are shown. * *p* < 0.05, ** *p* < 0.01 and **** *p* < 0.0001.

To validate the 30GS in an independent clinical dataset and assess its association with treatment response, we analyzed pre-treatment tumor microarray data from the I-SPY2 trial cohort. Consistent with findings from the TCGA dataset, the 30GS differed significantly across breast cancer subtypes within I-SPY2, including HR+/HER2-, HR+/HER2+, HR-/HER2+, and TNBC (*p* < 0.0001), with TNBC tumors exhibiting the lowest expression levels (Figure 5A). Across TNBC molecular classifications, the 30GS significantly differed (*p* < 0.0001) between Lehmann TNBC classification subtypes (basal-like1 [BL1], basal-like2 [BL2], immunomodulatory [IM], luminal androgen receptor [LAR], and mesenchymal [M]), with the BL1 subtype demonstrating the lowest 30GS expression. Similarly, stratification using the Burstein TNBC classification (Basal-like immune-activated [BLIA], basal-like immune-suppressed [BLIS], luminal androgen receptor [LAR], and mesenchymal [M]) revealed significant differences across subtypes (*p* < 0.0001), with BLIA tumors exhibiting the lowest 30GS level. These findings indicate that reduced 30GS expression is enriched in aggressive and basal-like TNBC subtypes.

We next assessed whether 30GS expression was associated with pathological complete response (pCR) in TNBC patients. ROC analysis demonstrated that, among TNBC patients treated with paclitaxel alone (*n* = 85), the 30GS modestly predicted pCR (AUC 0.6325, *p* = 0.0205) (Figure 5B). In contrast, among patients treated with paclitaxel plus pembrolizumab (*n* = 29), predictive performance improved (AUC 0.7377, *p* = 0.0007), indicating the 30GS expression may be associated with response in the context of immunotherapy-treated TNBC. To benchmark these findings against established immunotherapy response predictors identified within I-SPY2, we compared ROC performance of the 30GS with predictors of response to immunotherapy, including ICS5, Chemokine12, Dendritic cell scores, Module 3 IFN score, Module 5 Tcell/Bcell score, and STAT1 [25]. In patients treated with paclitaxel plus pembrolizumab, 30GS demonstrated predictive performance (AUC 0.7377, *p* = 0.0007) comparable to ICS5 (AUC 0.7419, *p* = 0.0006), Chemokine12 (AUC 0.7920, *p* < 0.0001), Dendritic cell (AUC 0.7827, *p* < 0.0001), Module 5 Tcell/Bcell score (AUC 0.7547, *p* = 0.0003), and STAT1 (AUC 0.7835, *p* < 0.0001) (Figure 5C). As expected, Module3_IFN showed weaker predictive performance in this subset (AUC 0.5968, *p* = 0.1691). These comparisons demonstrate that the performance of the 30GS was comparable to that of previously reported immune-related signatures in the immunotherapy-treated TNBC cohorts.

To determine whether specific genes within the 30GS are associated with the observed immunotherapy-related signatures, we performed gene-by-gene correlation analyses against the strongest I-SPY2 predictors of immunotherapy response (ICS5, Chemokine12, Dendritic cell, Module 5 Tcell/Bcell score, and STAT1). Among all genes, DUSP5 emerged as the most strongly and consistently positively correlated gene across all predictors (Figure 5D). Consistent with the TCGA association analysis, this finding identifies DUSP5 as a candidate gene associated with immune-related signatures and immunotherapy-associated transcriptional programs in TNBC. Given that DUSP5, among the 30GS genes, demonstrated consistent associations with immune-related signatures and clinical outcomes in TNBC across the TCGA and I-SPY2 datasets, DUSP5 was therefore prioritized for further validation.

### 3.6. DUSP5-Associated Transcriptional Programs in TNBC Tumors

**Figure 6:**
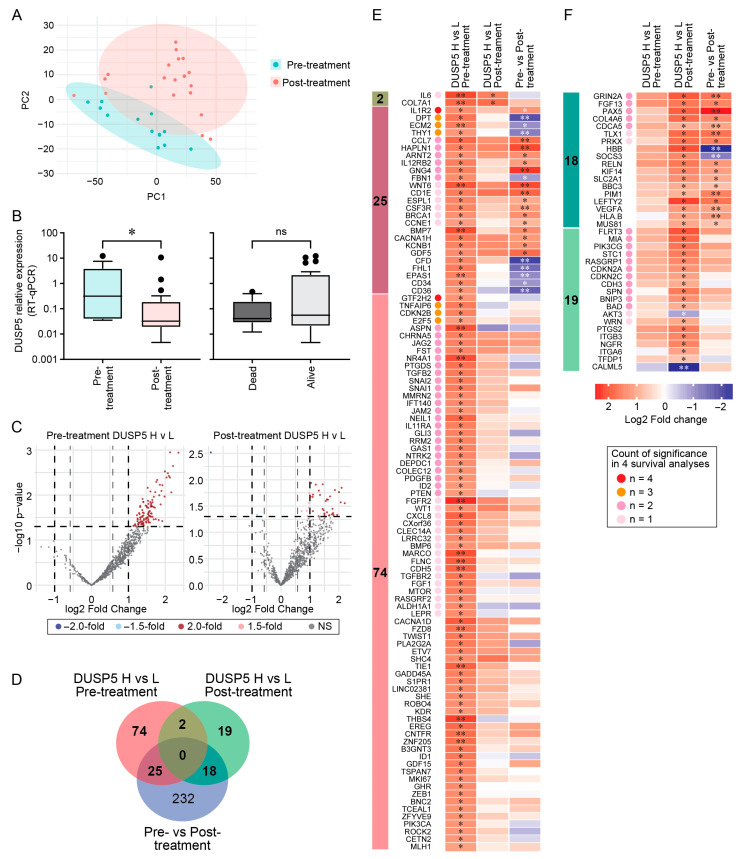
DUSP5-associated transcriptional program in an independent TNBC patient cohort. TNBC FFPE tumor samples comprising pre-treatment (*n* = 14) and post-treatment (*n* = 20) cohorts were analyzed for DUSP5 expression and NanoString Breast Cancer (BC360) panel gene expression. (**A**) Principal component analysis (PCA) of pre-treatment and post-treatment tumors based on BC360 gene expression profiles. Each point represents an individual patient projected onto the first two principal components (PC1 and PC2). Shaded ellipses represent the 95% confidence intervals for each group. (**B**) Relative DUSP5 expression measured by RT-qPCR in pre-treatment and post-treatment tumors, and in alive versus dead patients. DUSP5 expression is presented as relative expression normalized to housekeeping genes. ns = not significant, * *p* < 0.05, unpaired Student’s *t*-test. (**C**) Volcano plots showing differential gene expression between DUSP5-high (DUSP5 H) and DUSP5-low (DUSP5 L) tumors in the pre-treatment and post-treatment cohorts. Significantly upregulated genes are shown in red, and non-significant genes in grey. (**D**) Venn diagram summarizing overlap of differentially expressed genes identified in pre-treatment versus post-treatment tumors, DUSP5 H versus DUSP5 L tumors in the pre-treatment cohort, and DUSP5 H versus DUSP5 L tumors in the post-treatment cohort. Differential expression analysis comparing pre-treatment and post-treatment tumors identified 275 significantly dysregulated genes (Supplementary Figure S3A). (**E**,**F**) Heatmaps of the 101 and 37 differentially expressed genes identified between DUSP5 H and DUSP5 L tumors in the pre-treatment (**E**) and post-treatment (**F**) cohorts, respectively. Two genes overlapped between the pre-treatment and post-treatment DUSP5-associated gene sets. Heatmap values represent log2 fold change from differential expression analyses, with significance indicated by asterisks (* *p* < 0.05, ** *p* < 0.01; limma, data provided as Supplementary Table S2). Genes marked with dots demonstrated significant association with survival in four independent survival analyses (Supplementary Figure S3B,C). Dot color corresponds to the number of significant survival associations. Left side-annotation bars indicate gene grouping based on overlap categories shown in panel (**D**).

We used an independent TNBC cohort comprising pre-treatment (*n* = 14) and post-treatment (*n* = 20) tumor samples to investigate the association of DUSP5 expression with genes in the NanoString Breast Cancer 360 (BC360) panel. Principal component analysis (PCA) of BC360 gene expression profiles demonstrated clear segregation between pre-treatment and post-treatment samples (Figure 6A). As expected, we observed transcriptional remodeling following neoadjuvant chemotherapy. Accordingly, subsequent analyses were performed independently within each subset to account for treatment-related transcriptional differences.

Relative DUSP5 gene expression measured by RT-qPCR was significantly lower in post-treatment than pre-treatment tumors (*p* = 0.0171) and lower in dead than in alive patients, although this difference was not statistically significant (*p* = 0.1836) (Figure 6B). To analyze the BC360 panel genes in the context of DUSP5 expression, patients were dichotomized into DUSP5-High (DUSP5 H) and DUSP5-Low (DUSP5 L) groups based on the upper and lower 50% of DUSP5 expression, respectively. Differential expression analyses comparing DUSP5 H and DUSP5 L tumors identified 101 significantly upregulated genes in the pre-treatment subset, and 39 significantly upregulated genes in the post-treatment subset using a threshold of ≥1.5-fold change (log2 ≥ 0.58) and *p* < 0.05 (Figure 6C). These genes were subsequently intersected with genes differentially expressed between pre-treatment and post-treatment tumors (Figure 6D; a volcano plot for differential expression comparing pre-treatment and post-treatment tumors is provided as Supplementary Figure S3A). Two genes (*IL6* and *COL7A1*) were consistently associated with DUSP5 status in both pre-treatment and post-treatment tumors, independent of treatment effects, whereas 99 genes and 37 genes were uniquely associated with DUSP5 status in the pre-treatment and post-treatment samples, respectively, yielding a total of 138 DUSP5-associated genes across both treatment groups.

Survival association analyses were performed for the 138 DUSP5-associated genes using four complementary survival approaches: differential expression analyses comparing tumors from alive versus dead patients in pre-treatment and post-treatment subsets (Supplementary Figure S3B), and Cox proportional hazards regression analyses within the pre-treatment and post-treatment subsets (Supplementary Figure S3C). Among the two treatment-independent DUSP5-associated genes, IL6 was additionally associated with survival. Fold-expression changes and survival associations across the four analyses are presented as heatmap visualizations of the 101 and 37 differentially expressed genes identified in the pre-treatment (Figure 6E) and post-treatment (Figure 6F) tumors, respectively. Individual expression profiles of the 138 DUSP5-associated genes stratified by DUSP5 status and treatment condition are provided in Supplementary Figure S4.

**Figure 7:**
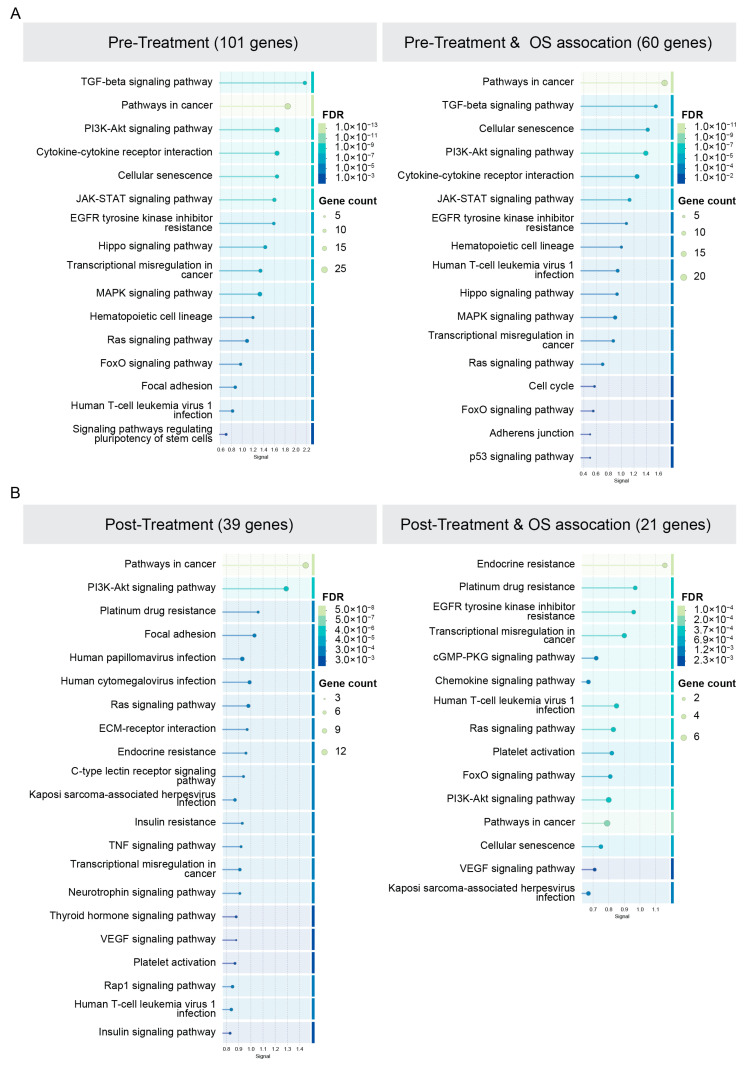
KEGG pathway analysis of DUSP5-associated genes. (**A**) KEGG pathway analysis of 101 DUSP5-associated genes identified in the pre-treatment cohort and their survival-associated subset (60 genes). (**B**) KEGG pathway analysis of the 39 DUSP5-associated genes identified in the post-treatment cohort and their survival-associated subset (21 genes). Pathway analysis was performed using STRING-DB (https://string-db.org/) pathway annotation. The Top 30 enriched pathways are provided in Supplementary Figure S5. Pathways with broad or non-informative annotations were excluded from the main figure for visualization clarity. As the NanoString BC360 panel presents a pre-selected pathway-focused gene set, enrichment statistics should be interpreted within the context of targeted panel design.

KEGG pathway analysis performed on STRING-DB using the 101 DUSP5-associated genes identified in the pre-treatment tumors and their survival-associated subset (60 genes) demonstrated enrichment of TGF-beta, PI3K-AKT, JAK-STAT, and MAPK signaling pathways, as well as cytokine-cytokine receptor interaction pathways (Figure 7A, the top 30 enriched pathways are provided as Supplementary Figure S5). Furthermore, KEGG pathway analysis of the 39 DUSP5-associated genes identified in the post-treatment tumors (two of which overlapped with genes identified in the pre-treatment subset) and their survival-associated subset (21 genes) demonstrated enrichment of PI3K-AKT, Ras, TNF, and chemokine signaling pathways (Figure 7B).

## 4. Discussion

In this study, we identified an EZH2-associated epigenetically repressed transcriptional program that distinguishes triple-negative breast cancer (TNBC) from other breast cancer subtypes and is linked to tumor-intrinsic immune-related programs and to response to immunotherapy. Through integrative analysis of the TCGA breast cancer cohort, we defined a 30-gene signature (30GS) characterized by transcriptional repression and promoter hypermethylation, associated with elevated EZH2 expression. Reduced expression of this signature was enriched in TNBC and basal-like tumors and was associated with poorer clinical outcomes, particularly disease-free intervals, progression-free intervals, and distant metastasis-free survival. The 30GS retained independent prognostic value beyond standard clinicopathological variables, suggesting that it captures tumor-intrinsic features not reflected by traditional clinical parameters. While survival analyses were performed in the overall breast cancer cohort to ensure an unbiased representation of the 30GS, the relevance of the 30GS is further supported by its enrichment in TNBC tumors and by consistent immune associations with TNBC-specific analyses.

The 30GS showed consistent associations with immune-related pathways, including T-cell activation, interferon signaling, and immune checkpoint expression, features characteristic of an immune-inflamed tumor phenotype associated with responsiveness to immune checkpoint blockade [28,29]. While T-cell exhaustion is characterized by sustained expression of inhibitory receptors, exhausted T-cells frequently coexist with activated T-cell states within inflamed tumors [30,31]. Accordingly, the association of the 30GS with both activation and checkpoint-related features suggests that it reflects a broader immune-inflamed state rather than a purely exhausted phenotype.

The clinical relevance of the 30GS lies in its ability to capture tumor-intrinsic epigenetic states that are not reflected by current immune-based biomarkers. While biomarkers such as PD-L1 expression and tumor-infiltrating lymphocytes provide insight into the immune composition of the tumor microenvironment, they do not capture the underlying tumor-intrinsic programs that regulate immune engagement. In this context, the 30GS may serve not only as a prognostic indicator but also as a surrogate marker of tumor-intrinsic immune competence. This may have contributed to the stronger performance of the 30GS in the immunotherapy-treated I-SPY2 cohort. Nevertheless, it remains unclear whether this reflects a treatment-specific predictive effect or a more general association with favorable tumor-intrinsic immune states. Consistent with previously reported signatures in I-SPY2 [25], these findings likely reflect baseline tumor-intrinsic immune states, and further validation will be required to establish predictive specificity.

The consistent performance of the 30GS across independent datasets, despite differences in platform and gene availability, supports its robustness as a composite measure of coordinated gene expression. Notably, several genes within the 30GS are associated with hormone receptor signaling and are expected to be expressed at lower levels in TNBC. However, their reduced expression is accompanied by promoter hypermethylation and association with EZH2 expression, suggesting that this pattern is not solely attributable to lineage-specific biology. Instead, these findings support a model in which EZH2-associated epigenetic repression contributes to downregulation of the 30GS in TNBC alongside subtype-related transcriptional programs.

Although promoter hypermethylation is generally associated with transcriptional repression, gene expression is regulated by multiple interconnected mechanisms, including histone modifications, transcription factor activity, chromatin accessibility, and post-transcriptional regulation [32]. As a result, not all genes exhibit a strict inverse relationship between methylation and expression. The observed variability likely reflects the complexity of epigenetic and transcriptional regulation within the tumor microenvironment. In this context, EZH2-mediated repression may act in concert with other chromatin-modifying processes, suggesting that the 30GS reflects a broader epigenetic regulatory state rather than a single mechanism. However, our findings suggest that EZH2-associated epigenetic remodeling may represent a tumor-intrinsic state associated with the immune landscape of TNBC and immunotherapy-related transcriptional programs.

A key insight from this study is the identification of DUSP5 as a candidate linked to EZH2-mediated epigenetic regulation and immune-related transcriptional programs in TNBC. While the role of DUSP5 in tumor immunity remains largely unexplored, MAPK/ERK signaling, which is negatively regulated by DUSP5, is known to influence key immune-related processes, including modulation of interferon signaling, cytokine production, and immune checkpoint expression [33]. In this context, analysis of an independent TNBC cohort further supported the association between DUSP5 expression and immune-related transcriptional programs. Although neoadjuvant chemotherapy induced transcriptional remodeling, DUSP5-associated immune-related transcriptional programs remained evident across treatment contexts, suggesting that these relationships are not solely treatment-driven. Importantly, a substantial proportion of DUSP5-associated genes were additionally linked to survival, further supporting the clinical relevance of DUSP5-associated transcriptional states in TNBC. Pathway analyses demonstrated enrichment of inflammatory and immune-related pathways, including PI3K/AKT, JAK/STAT, MAPK, cytokine-cytokine receptor interaction pathways, chemokine signaling, TNF signaling, and TGF-beta signaling pathways, consistent with an immune-related transcriptional phenotype. Emerging evidence suggests that tumor intrinsic pathways such as PI3K/AKT, JAK/STAT, and MAPK may influence anti-tumor immunity through the modulation of cytokine signaling, antigen presentation, and immune checkpoint regulation, further supporting the interplay between oncogenic signaling and immune remodeling in TNBC [34].

Furthermore, IL6 emerged as one of the treatment-independent DUSP5-associated genes linked to survival, suggesting a potential interaction between DUSP5-associated transcriptional programs and inflammatory signaling networks in TNBC. IL6 is a pleiotropic inflammatory cytokine with established roles in tumor-immune communication, cytokine-mediated signaling, and therapeutic responsiveness [35]. Dysregulated IL6 signaling has been implicated in inflammatory remodeling and resistance to therapy in TNBC and other malignancies [36]. Collectively, these findings provide independent validation from patient cohorts, supporting the association between elevated DUSP5 expression and an inflamed tumor microenvironment.

Several limitations should be considered. The present study provides strong correlative and translational evidence derived from independent transcriptomic datasets; however, these findings do not establish causality. In particular, the observed associations among EZH2 expression, promoter hypermethylation, and gene expression reflect associations rather than direct mechanistic proof. Furthermore, while DUSP5 was prioritized based on consistent associations with immune-related signatures, immunotherapy response predictors, and clinical outcomes across independent datasets, its functional role in modulating MAPK signaling and tumor-immune interactions in TNBC remains to be fully elucidated. DUSP5 is a known regulator of MAPK/ERK signaling, which is implicated in both immune modulation and tumor progression, providing a plausible biological link between DUSP5 expression and immune-related phenotypes. However, the current findings are primarily correlative, and functional validation, including gene perturbation studies, will be required to establish a causal role for DUSP5 in regulating tumor-immune interactions and therapeutic response in TNBC. Finally, the immunotherapy prediction analysis was performed in a relatively small subgroup of patients treated with chemotherapy plus pembrolizumab, which may limit statistical power and generalizability. Therefore, these findings should be interpreted with caution, and further validation in larger prospective cohorts will be required to determine the clinical utility of the 30GS as a predictive biomarker for immunotherapy response in TNBC, including the establishment of clinically relevant cutoff thresholds.

## 5. Conclusions

In summary, this study identifies an EZH2-associated epigenetically repressed gene signature linked to tumor-intrinsic immune-related programs in TNBC. These findings support further investigation of epigenetic biomarkers for patient stratification and therapeutic response assessment in TNBC. The identification of DUSP5 further highlights a candidate pathway associated with immune-related transcriptional programs, warranting additional mechanistic and translational investigation in the context of immune checkpoint blockade.

## Data Availability

The datasets analyzed in this study are publicly available. TCGA breast cancer data are available through the Genomic Data Commons (GDC) portal. I-SPY2 data are available in the Gene Expression Omnibus (GEP) under accession number GSE194040. Additional data generated in this study are available from the corresponding author upon request.

## Supplementary Materials

The following supporting information can be downloaded at: https://www.mdpi.com/article/10.3390/cancers18101606/s1, Figure S1: DNA methylation analysis of EZH2-target genes in TNBC. Figure S2: Multivariable Cox proportional hazards analysis of the 30-gene signature (30GS) across clinical endpoints. Figure S3: Survival-associated analyses of DUSP5-associated transcriptional programs in the independent TNBC cohort. Figure S4: Expression profiles of DUSP5-associated genes across treatment and DUSP5 expression groups (legend provided separately as Legend S4) Figure S5: KEGG pathway analysis of DUSP5-associated genes in the independent TNBC cohort. Table S1: Summary of clinicopathological characteristics of the indepenent TNBC cohort used for NanoString Breast Cancer 360 (BC360) panel profiling. Table S2: Data of differential expression and survival-associated analyses of DUSP5-associated genes in the indepenent TNBC cohort.

## Abbreviations

The following abbreviations are used in this manuscript:

TNBC	Triple-Negative Breast Cancer
TCGA	The Cancer Genome Atlas
DUSP5	Dual Specificity Phosphatase 5
EZH2	Enhancer of Zeste Homolog 2
