# Supplementary material for: EZH2-Associated Hypermethylated Gene Signature Predicts Immunotherapy Response and Implicates DUSP5 in Tumor-Immune Regulation in Triple-Negative Breast Cancer
Source: Cancers (Basel). 2026 May 15;18(10):1606. doi: 10.3390/cancers18101606 (PMC13204126; doi:10.3390/cancers18101606)
Supplement: Supplementary file 1 [file cancers-18-01606-s001.zip › Figure S4.pdf]

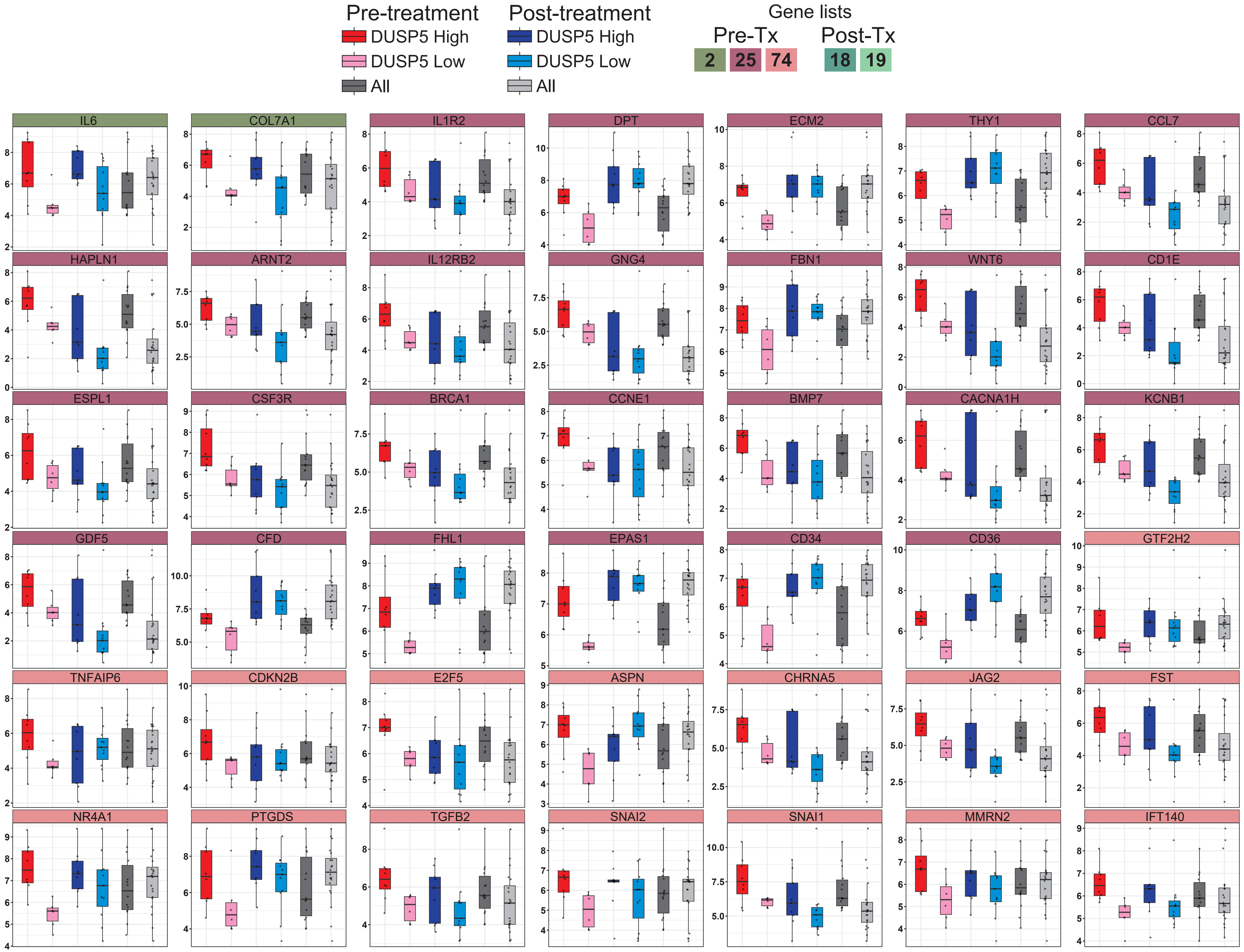
Pre-treatment
DUSP5 High
DUSP5 Low
All
Post-treatment
DUSP5 High
DUSP5 Low
All
Gene lists
Pre-Tx
2
25
74
Post-Tx
18
19
IL6
COL7A1
IL1R2
DPT
ECM2
THY1
CCL7
HAPLN1
ARNT2
IL12RB2
GNG4
FBN1
WNT6
CD1E
ESPL1
CSF3R
BRCA1
CCNE1
BMP7
CACNA1H
KCNB1
GDF5
CFD
FHL1
EPAS1
CD34
CD36
GTF2H2
TNFAIP6
CDKN2B
E2F5
ASPN
CHRNA5
JAG2
FST
NR4A1
PTGDS
TGFB2
SNAI2
SNAI1
MMRN2
IFT140

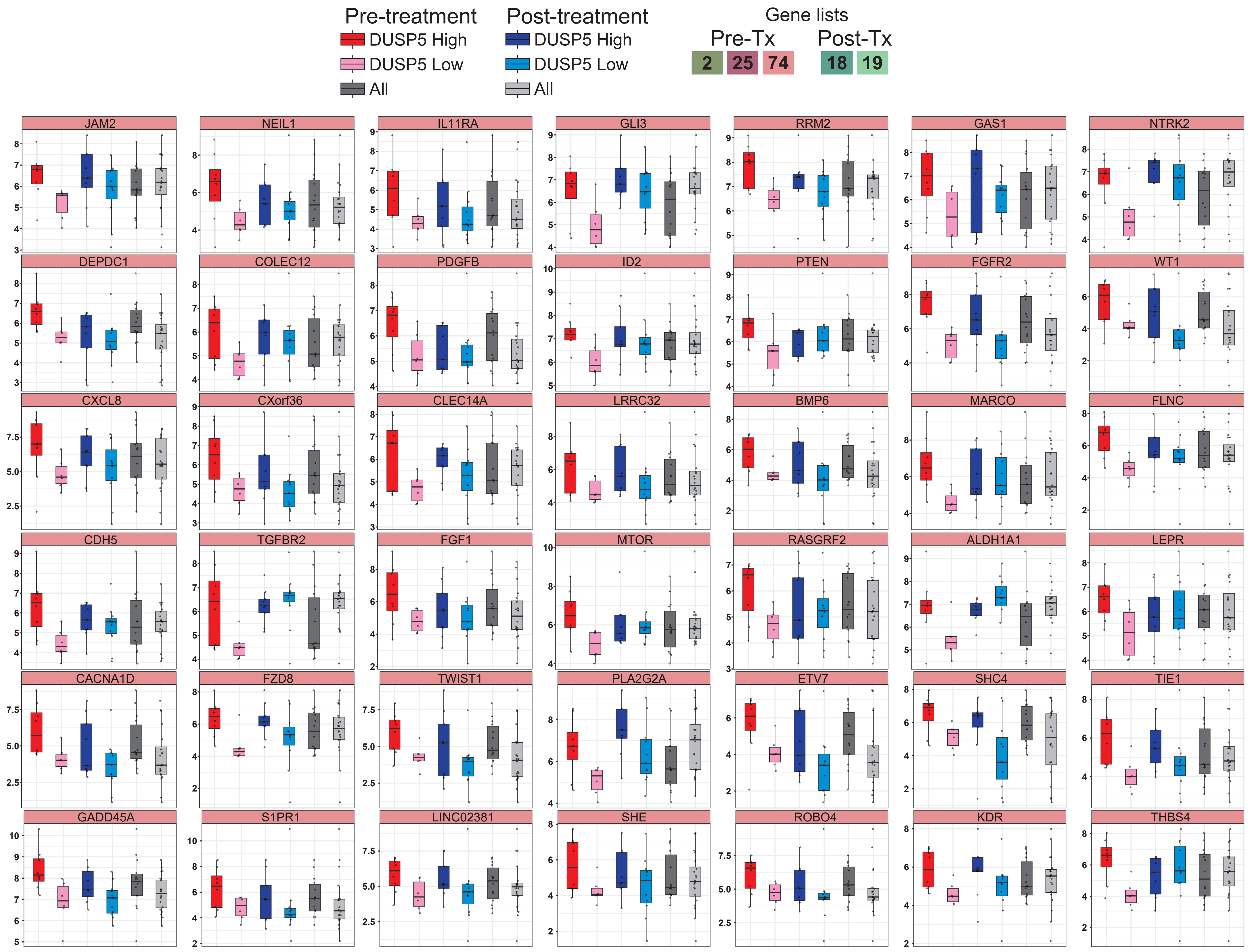

Pre-treatment
DUSP5 High
DUSP5 Low
All
Post-treatment
DUSP5 High
DUSP5 Low
All
Gene lists
Pre-Tx
2
25
74
Post-Tx
18
19
JAM2
NEIL1
IL11RA
GLI3
RRM2
GAS1
NTRK2
DEPDC1
COLEC12
PDGFB
ID2
PTEN
FGFR2
WT1
CXCL8
CXorf36
CLEC14A
LRRC32
BMP6
MARCO
FLNC
CDH5
TGFBR2
FGF1
MTOR
RASGRF2
ALDH1A1
LEPR
CACNA1D
FZD8
TWIST1
PLA2G2A
ETV7
SHC4
TIE1
GADD45A
S1PR1
LINC02381
SHE
ROBO4
KDR
THBS4

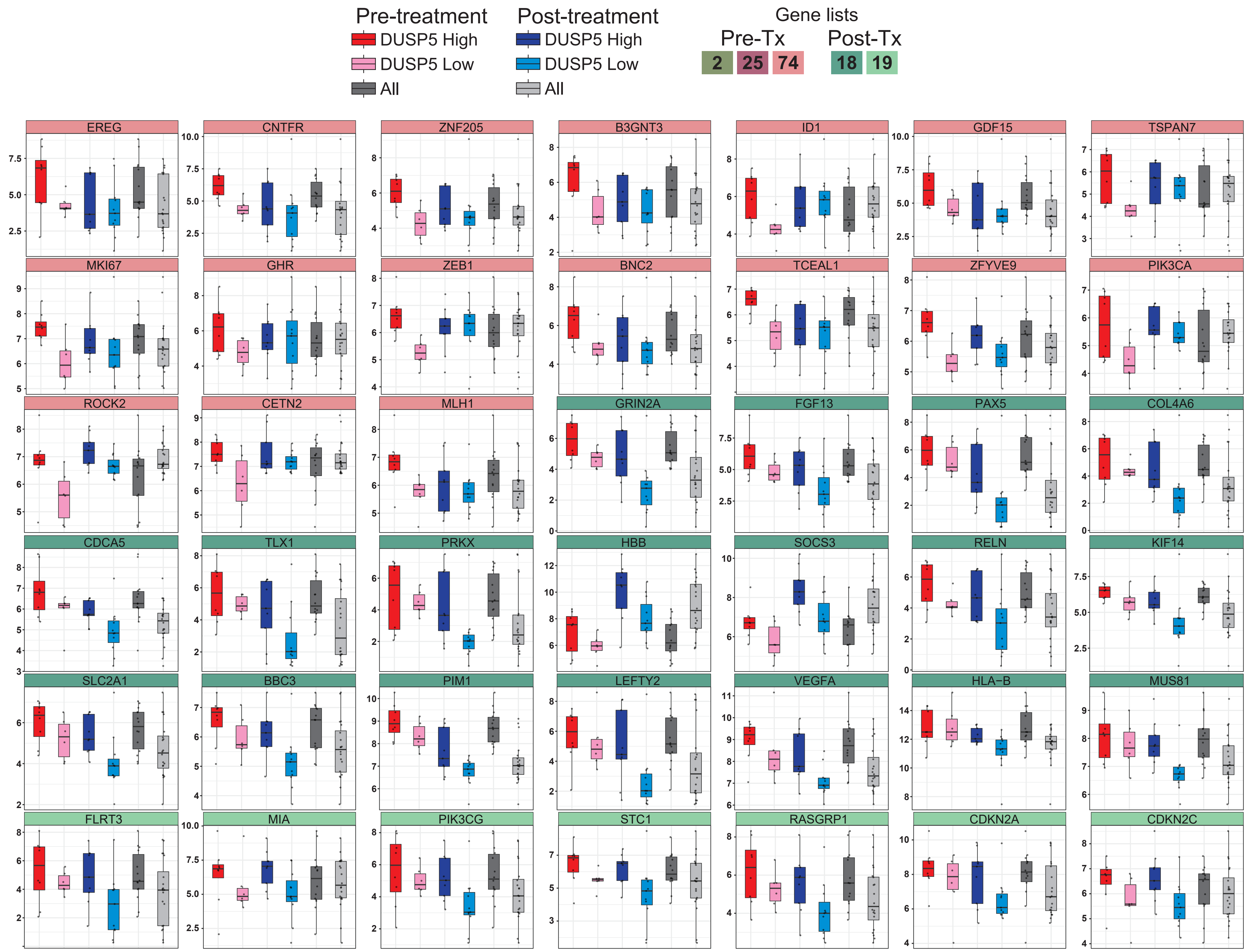
Pre-treatment
DUSP5 High
DUSP5 Low
All
Post-treatment
DUSP5 High
DUSP5 Low
All
Gene lists
Pre-Tx
2
25
74
Post-Tx
18
19
EREG
CNTFR
ZNF205
B3GNT3
ID1
GDF15
TSPAN7
MKI67
GHR
ZEB1
BNC2
TCEAL1
ZFYVE9
PIK3CA
ROCK2
CETN2
MLH1
GRIN2A
FGF13
PAX5
COL4A6
CDCA5
TLX1
PRKX
HBB
SOCS3
RELN
KIF14
SLC2A1
BBC3
PIM1
LEFTY2
VEGFA
HLA−B
MUS81
FLRT3
MIA
PIK3CG
STC1
RASGRP1
CDKN2A
CDKN2C

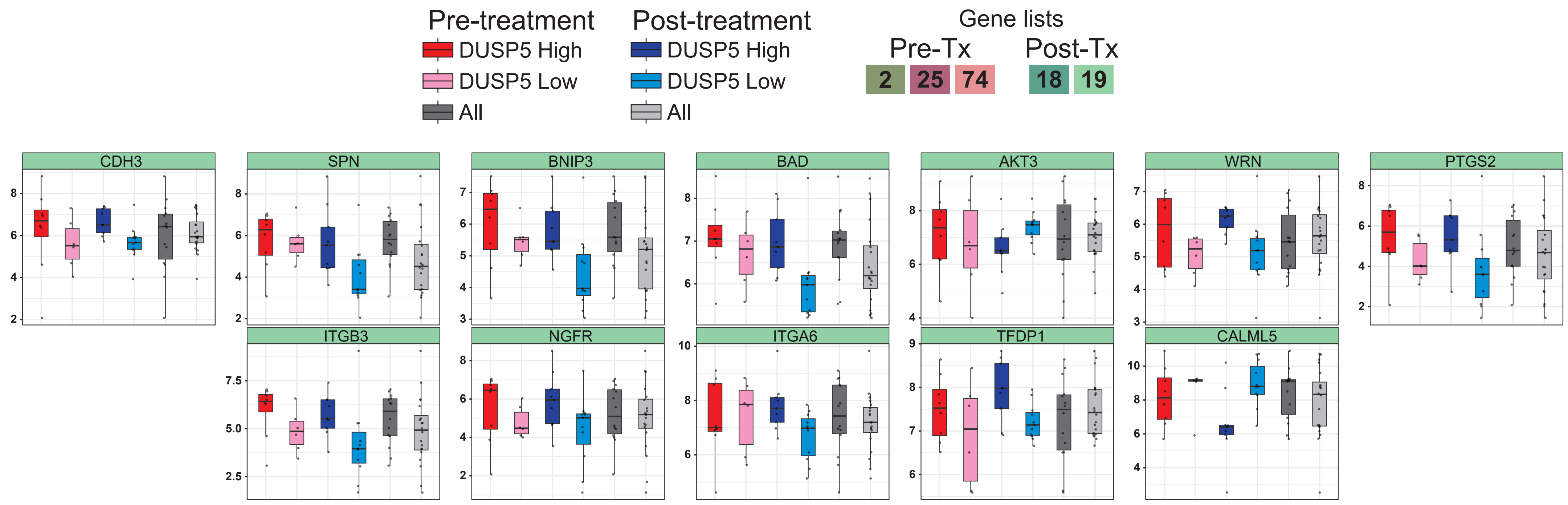
Pre-treatment
DUSP5 High
DUSP5 Low
All
Post-treatment
DUSP5 High
DUSP5 Low
All
Gene lists
Pre-Tx
2
25
74
Post-Tx
18
19
CDH3
SPN
BNIP3
BAD
AKT3
WRN
PTGS2
ITGB3
NGFR
ITGA6
TFDP1
CALML5
