# Supplementary figures and images for: EZH2-Associated Hypermethylated Gene Signature Predicts Immunotherapy Response and Implicates DUSP5 in Tumor-Immune Regulation in Triple-Negative Breast Cancer
Source: Cancers (Basel). 2026 May 15;18(10):1606. doi: 10.3390/cancers18101606 (PMC13204126; doi:10.3390/cancers18101606)

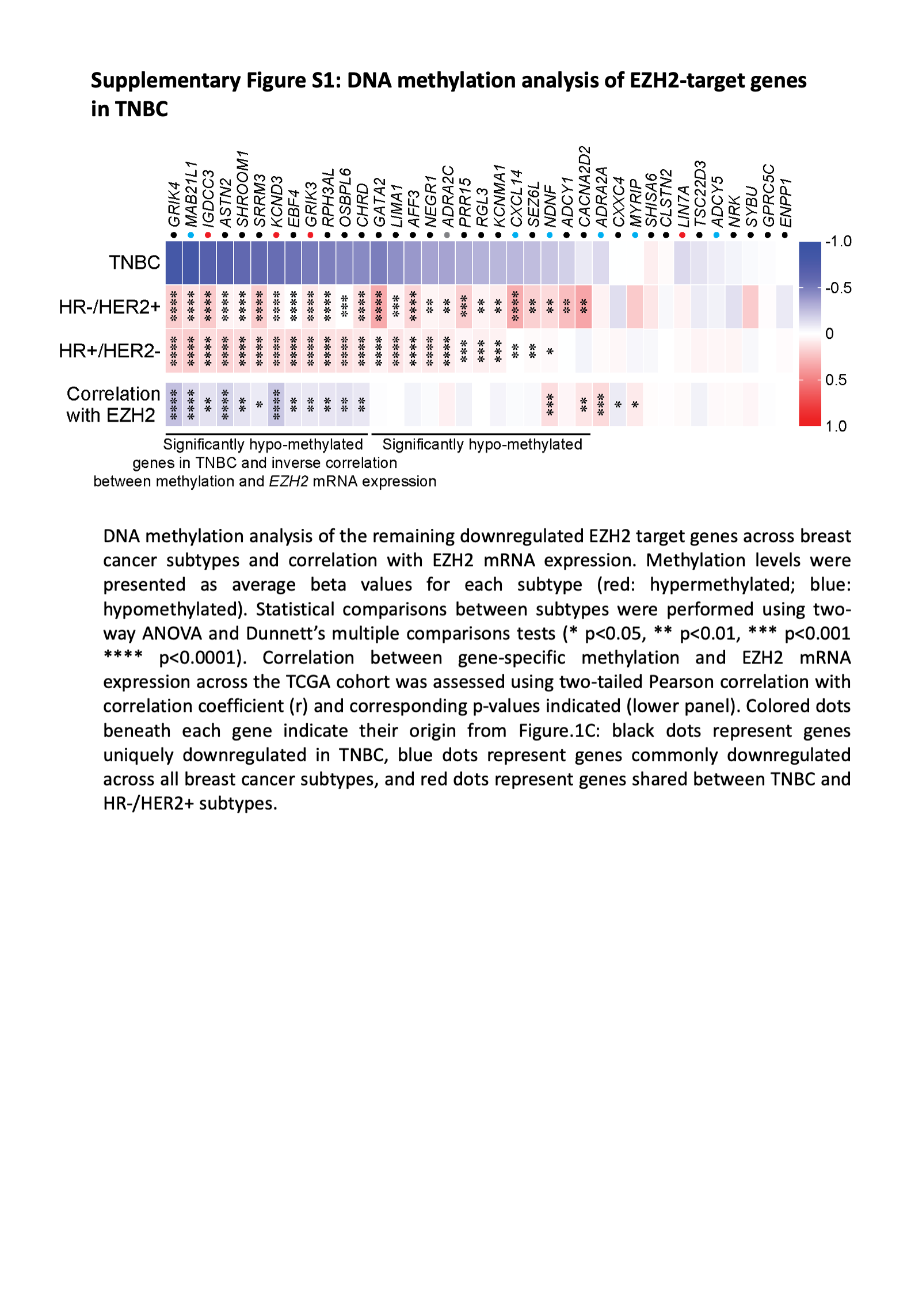

Supplement: Supplementary file 1 [file cancers-18-01606-s001.zip › Figure S1.tiff]

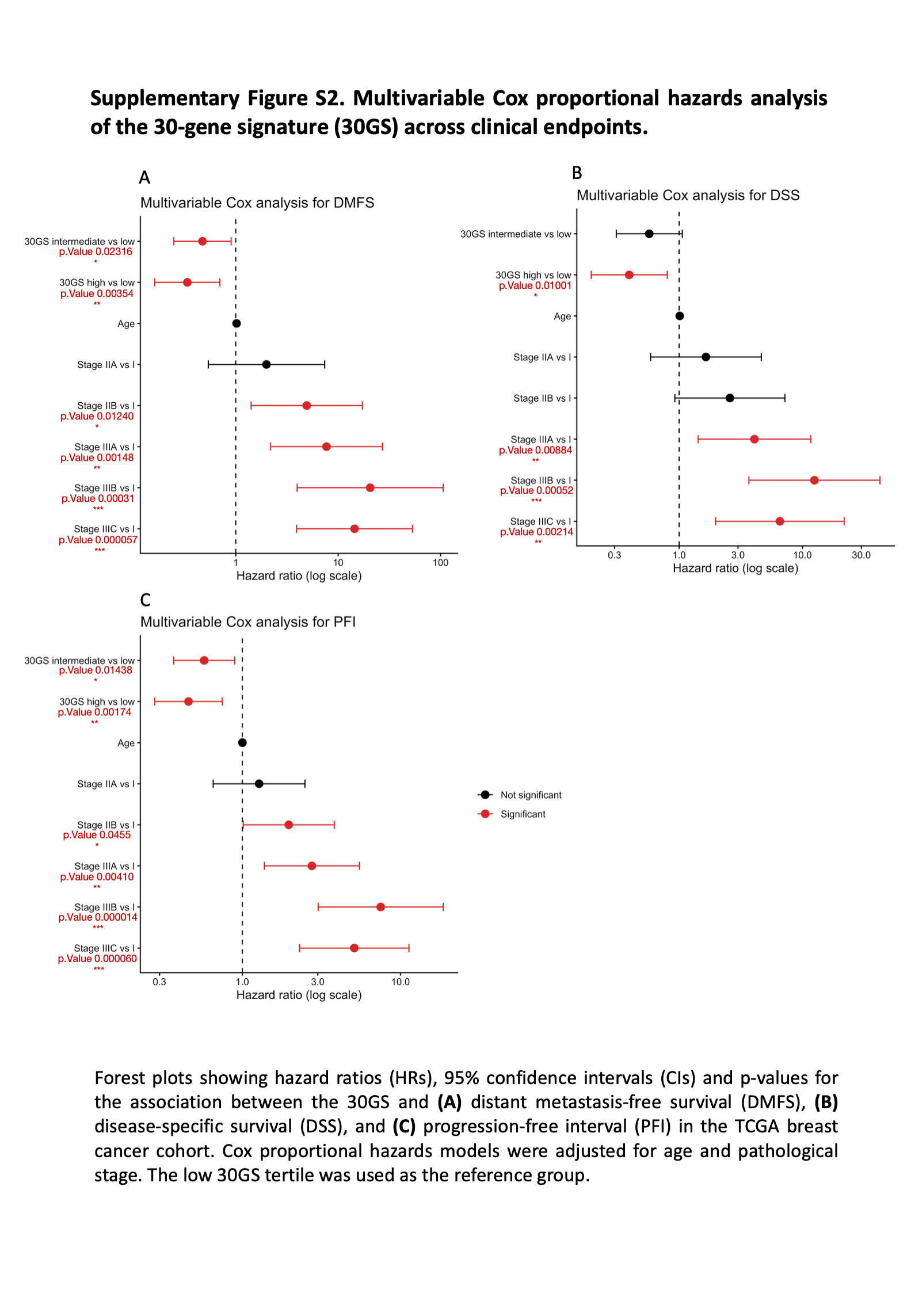

Supplement: Supplementary file 1 [file cancers-18-01606-s001.zip › Figure S2.tiff]

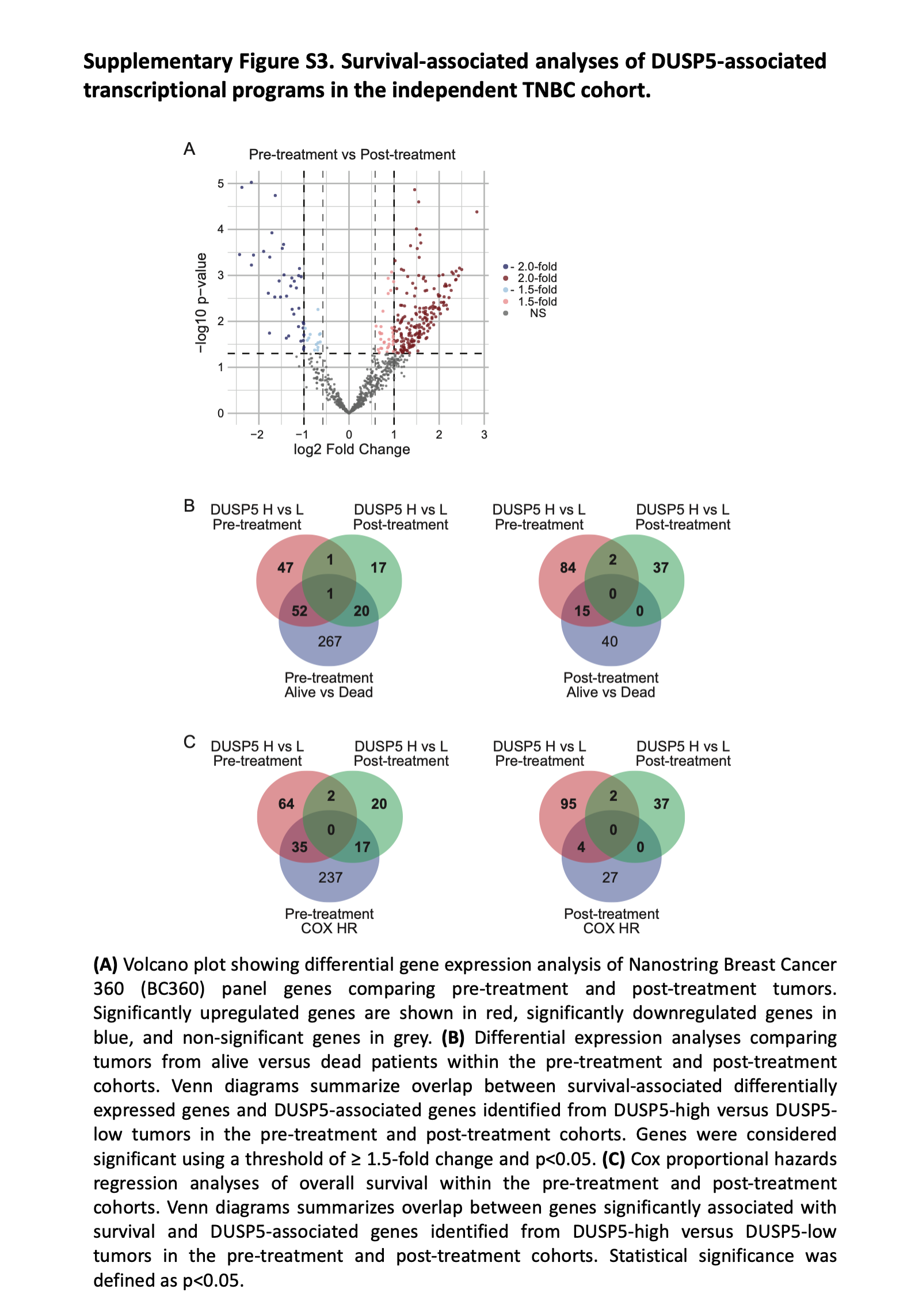

Supplement: Supplementary file 1 [file cancers-18-01606-s001.zip › Figure S3.tiff]

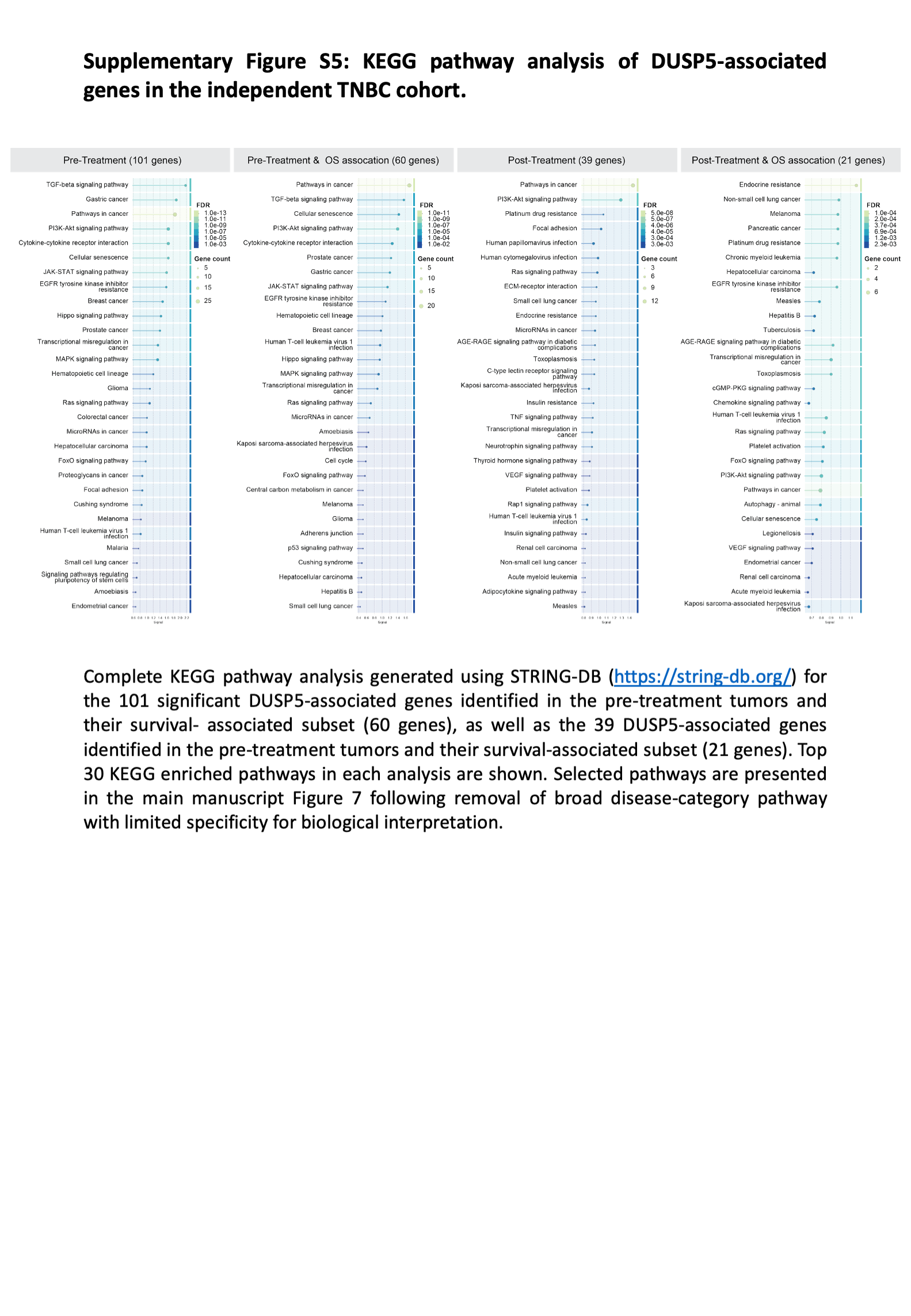

Supplement: Supplementary file 1 [file cancers-18-01606-s001.zip › Figure S5.tiff]

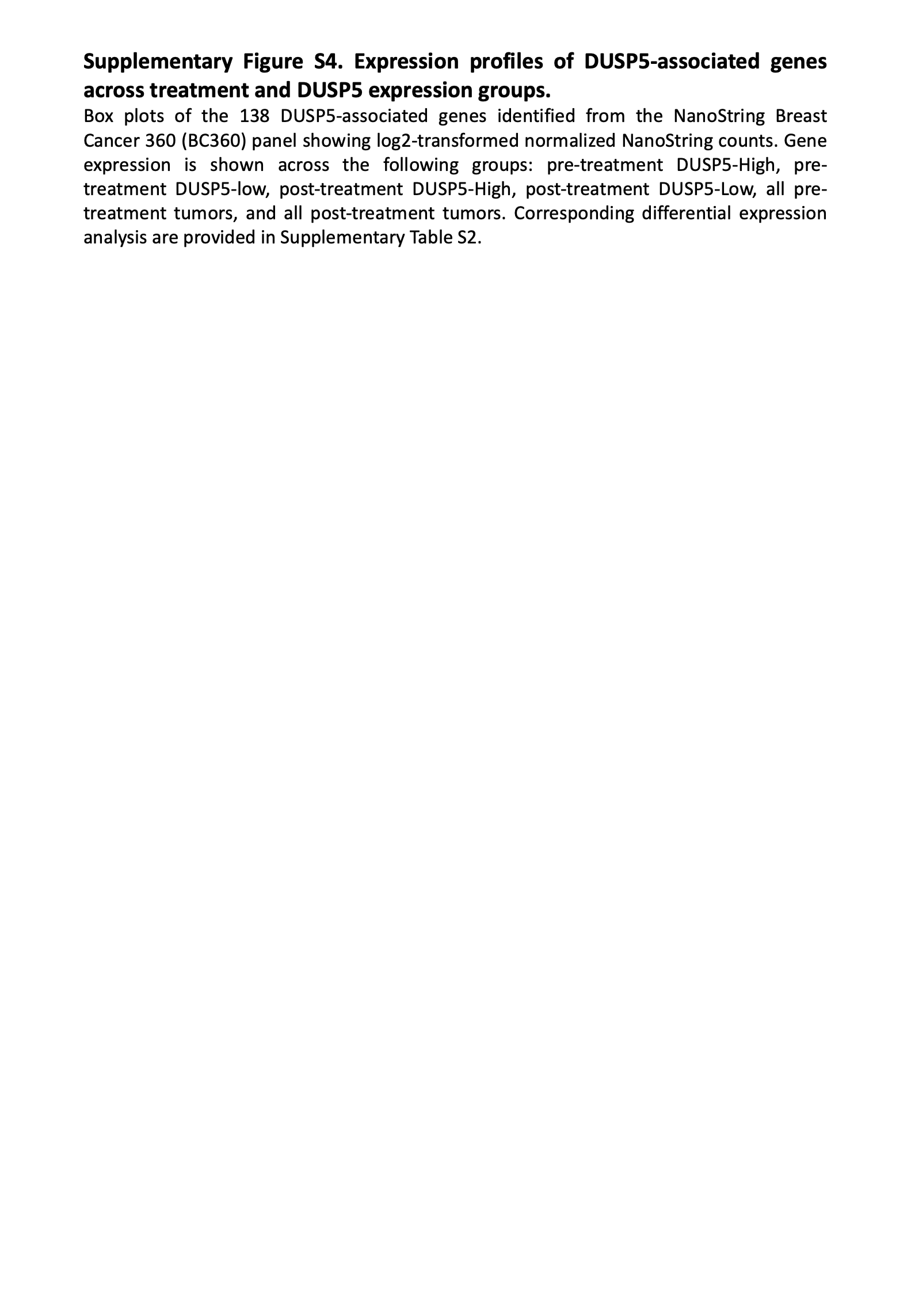

Supplement: Supplementary file 1 [file cancers-18-01606-s001.zip › Legend S4.tiff]

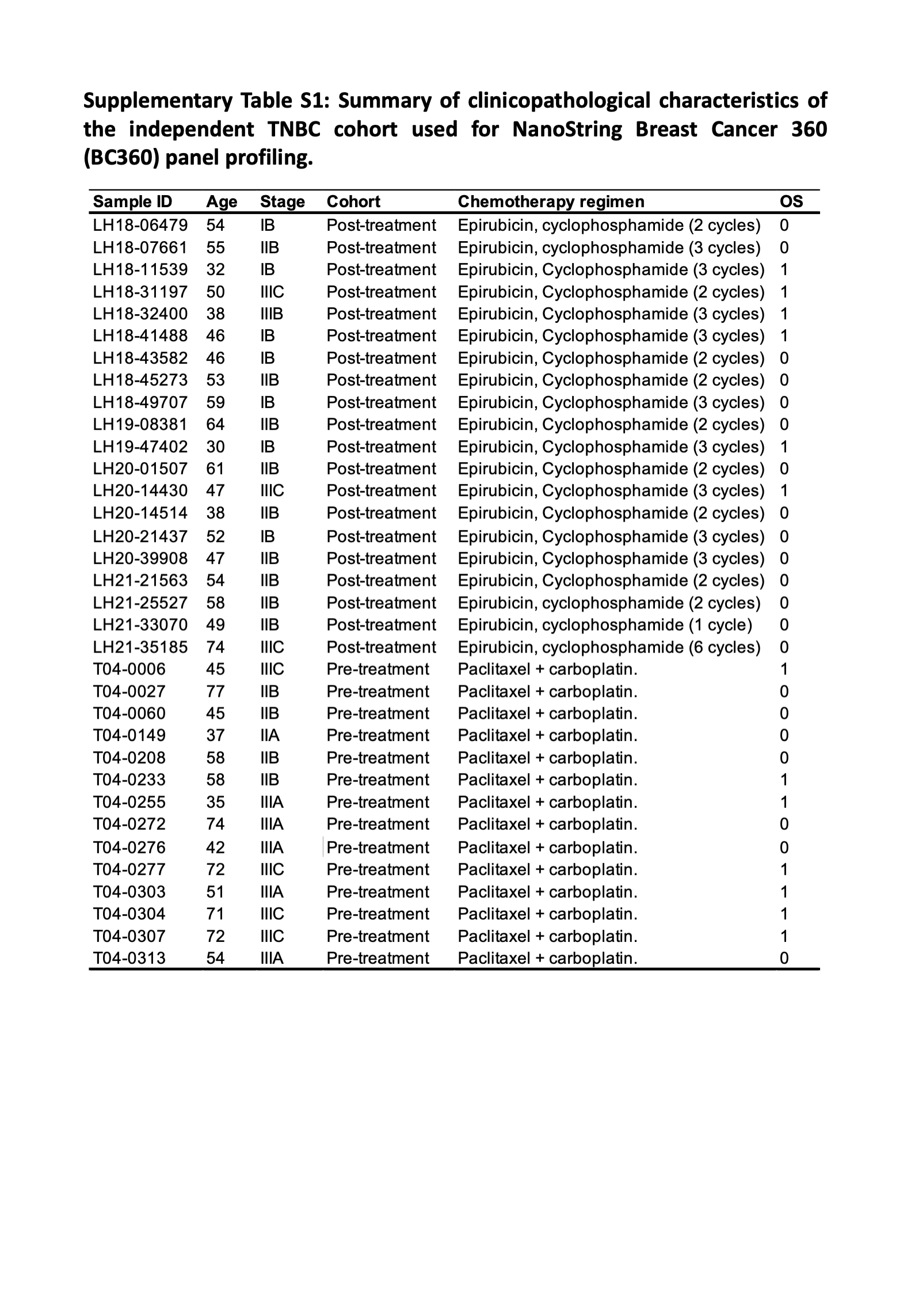

Supplement: Supplementary file 1 [file cancers-18-01606-s001.zip › Table S1.tiff]
